# The number of metastatic lymph nodes exhibiting poorly differentiated clusters predicts survival in patients with pStage III colorectal cancer

**DOI:** 10.1007/s00384-015-2393-5

**Published:** 2015-09-28

**Authors:** Osamu Kinoshita, Mitsuo Kishimoto, Yasutoshi Murayama, Yoshiaki Kuriu, Masayoshi Nakanishi, Chohei Sakakura, Eigo Otsuji, Akio Yanagisawa

**Affiliations:** Department of Surgical Pathology, Kyoto Prefectural University of Medicine, 465 Kajii-cho, Kamigyo-ku, Kyoto, 602-8566 Japan; Department of Surgery, Division of Digestive Surgery, Kyoto Prefectural University of Medicine, 465 Kajii-cho, Kamigyo-ku, Kyoto, 602-8566 Japan

**Keywords:** Colorectal cancer, Lymph node status, Poorly differentiated clusters, Histopathological prognosticator

## Abstract

**Purpose:**

Many studies have proposed alternative designations for lymph node (LN) status in colorectal cancer (CRC); however, knowledge of histopathological features in metastatic lymph nodes (MLNs) is limited. This study investigated the clinicopathological significance of poorly differentiated clusters (PDCs) in MLNs.

**Methods:**

Slides from 159 patients with pathological Stage III CRC were reviewed. Those with <12 dissected LNs (DLNs) were ineligible. PDCs composed of ≥5 cancer cells lacking full glandular formation and ≥10 PDCs under ×20 objective lens were defined as positive, and the number of MLNs with positive PDCs (MLNs-PDCs) was counted. Results were correlated with patient survival and comparisons made with other indications of LN status.

**Results:**

The mean numbers of MLNs and MLNs-PDCs were 2.8 and 1.0, respectively, and were moderately and positively correlated with each other. Univariate analysis identified cutoffs of ≥5 MLNs (86 vs. 55 %, *p* = 0.024), ≥2 MLNs-PDCs (85 vs. 63 %, *p* = 0.008), and ≥30 % LN ratio (85 vs. 44 %, *p* = 0.036) to indicate a positive LN status. However, no cutoff for DLNs was obtained. MLNs-PDCs (≥2) were associated with pT4 tumor (*p* = 0.0035), open surgery (*p* = 0.016), greater number of MLNs (*p* < 0.0001), and positive-PDC primary tumor (*p* < 0.0001). In multivariate analysis, a prognostic model incorporating ≥2 MLNs-PDCs provided the lowest Akaike information criterion value; consequently, both pT4 tumors (*p* < 0.001) and ≥2 MLNs-PDCs (*p* = 0.038) were revealed to be significant prognosticators.

**Conclusion:**

Results showed that applying the number of MLNs-PDCs could improve stratification in pStage III CRC and may be a valuable candidate for LN status.

## Introduction

The incidence of colorectal cancer (CRC) has increased gradually over the decades in developed countries, and CRC now is the third most-commonly diagnosed cancer [1]. Recent progress in active cytotoxic chemotherapy regimens has contributed to prolonged survival of patients with CRC [2, 3]. However, to reduce mortality, there is a growing need for early prediction of prognosis to further improve postoperative interventions.

Since Dukes [4] reported the histopathological grading of rectal cancer in 1937, accumulated evidence on CRC has confirmed the clinical importance of lymph node (LN) status. In particular, the number of metastatic LNs (MLNs) has been considered one of the most powerful predictors in CRC [5, 6] and guides the indication for adjuvant chemotherapy. On the other hand, many researchers have proposed alternatives to indicate LN status, including the total number of dissected LNs (DLNs) [7–10], LN ratio (LNR) [11–15], and extramural cancer deposits (EX) [16, 17]. However, to date, published studies rarely have focused on the histopathological characteristics of MLNs.

More recently, a novel histological grading system using poorly differentiated clusters (PDCs), which are composed of ≥5 cancer cells that present at the invasive front of the tumor and lack full glandular formation, has been highlighted as a histopathological prognosticator of CRC [18–20]. Presence of PDCs was reported to be strongly positively correlated with LN metastasis and poorer survival and, consequently, was expected to provide a complementary approach to the conventional pathological diagnostic process. However, despite increasing evidence of PDCs as prognostic indicators, no previous studies have focused on PDCs in MLNs and their correlation to PDCs in primary tumors. An understanding of the histopathological behavior of PDCs in MLNs may provide insights into the key process following LN metastasis. Therefore, this study expanded upon the PDC concept of evaluation for histopathological features of MLNs and aimed to clarify the clinicopathological importance of PDCs in MLNs in comparison with previously reported LN status.

## Patients and methods

### Patients

This study examined data on patients with pathological Stage III (pStage III) CRC who had undergone potentially curative resection and systematic en bloc LN dissection at a single institution from 2000 to 2012. Since laparoscopic surgery became widespread during that period, cases in this study were treated with either open or laparoscopic surgery. Following the recommendation of the Union for International Cancer Control (UICC) TNM classification [5] to eliminate possible staging migration, patients with <12 DLNs were ineligible for this study. In addition, although pelvic sidewall LN dissection, which means removal of internal iliac and obturator LNs, traditionally has been performed in Japan for locally advanced lower rectal cancer [21, 22], patients on whom that dissection method had been performed were excluded. Other exclusion criteria for this retrospective cohort included (1) synchronous or multiple cancers, (2) preoperative therapy (neoadjuvant chemotherapy and endoscopic resection), (3) cancers related to ulcerative colitis or Crohn’s disease, and (4) familial adenomatous polyposis or hereditary non-polyposis colorectal cancer. To be included for analysis, after resection, patients were required to have follow-up surveillance at 3-month intervals for 1 year, then every 6 months for 3 years, and yearly thereafter. Prior to the study’s initiation, approval to use patient data for research purposes was obtained at the institution.

### Specimens

Within an hour after resection, all regional LNs were manually retrieved from the en bloc resected specimens after visual observation and palpation. The process of sectioning specimens after formalin fixation was performed according to Japanese guidelines [6]. In particular, larger retrieved LNs were longitudinally cut through the hilus, and smaller ones were embedded in paraffin whole. Sections 3 μm thick were cut from paraffin-embedded specimens and mounted onto glass slides. After deparaffinization, slides were stained with conventional hematoxylin and eosin according to the standard procedure.

### Histopathological evaluation

**Fig. 1:**
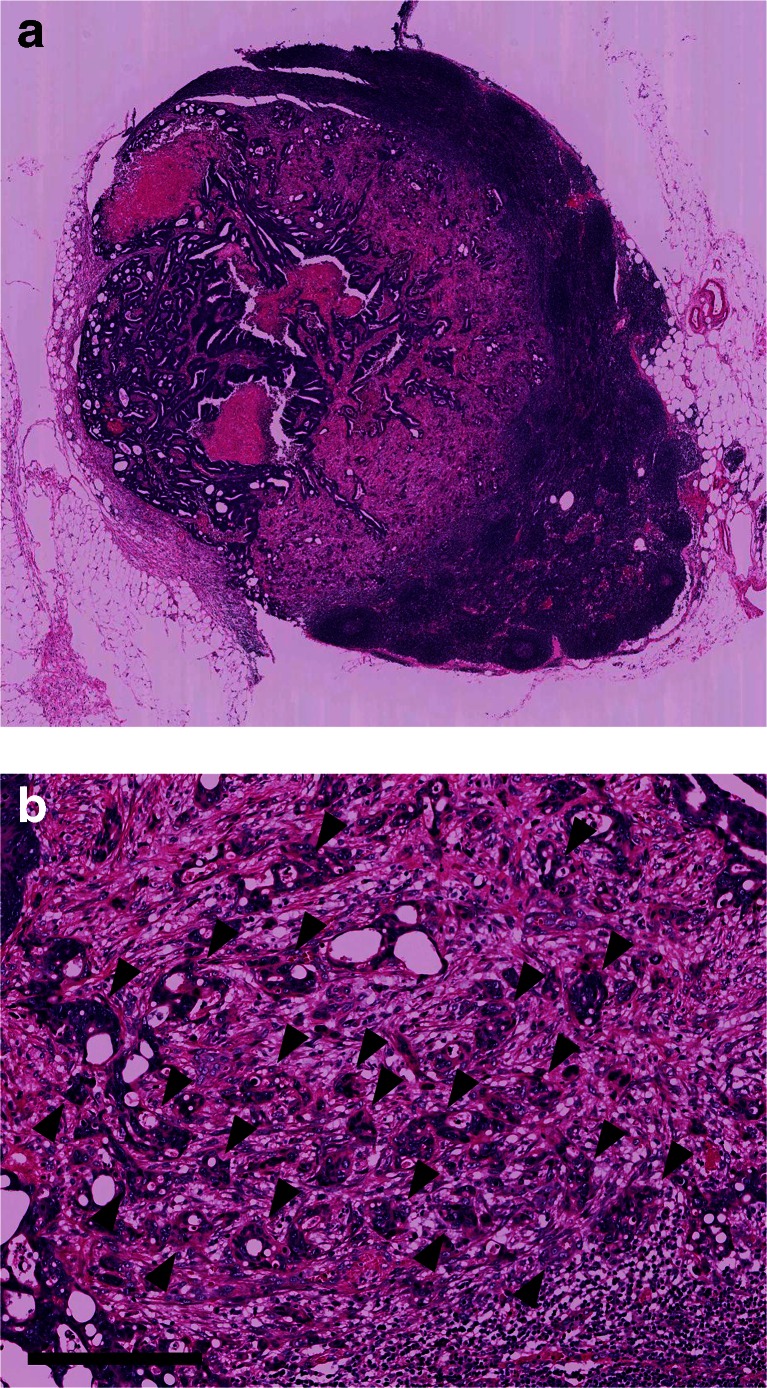
Representative examples of metastatic lymph nodes with positive PDCs under low-power view (**a**) and under the view of ×20 objective lens using hematoxylin and eosin stain (**b**). PDCs (>10) are observed (*arrowheads*). The *scale bar* indicates 200 μm

According to criteria set previously [18, 19], PDCs were defined as cancer clusters lacking full glandular formation and composed of ≥5 cancer cells, which are commonly observed at the invasive front of the tumor. All slides were reviewed pathologically, with special attention paid to the presence of PDCs, and ≥10 PDCs under a microscopic field with a ×20 objective lens were defined as positive PDCs. Our previous study [23] reported representative slides of PDCs in primary tumors. In the present study, PDCs in primary tumors were evaluated at the invasive front, whereas PDCs in MLNs were evaluated where they were observed most commonly (Fig. 1), and the number of MLNs with positive PDCs (MLNs-PDCs) was counted.

In addition to a pathologically proven number of DLNs, the LNR, defined as the number of MLNs divided by the number of DLNs, was assessed as LN status. For EX lacking residual LN structure, the study adopted the definition in Japanese guidelines [6]; consequently, EX were comprehensively considered as MLNs. However, tumor nodules that might be related to an isolated cancer focus in the lymphovascular vessels or perineural space were not considered as MLNs [16].

In addition, other pathological variables were obtained by observing slides. All slides were evaluated by a trained pathologist specializing in gastrointestinal pathology and blinded to the patients’ outcome.

### Statistical analysis

Overall survival (OS) curves were drawn by the Kaplan-Meier method, and comparisons between groups were performed using the log-rank test, with the length of the follow-up period being truncated at 5 years. In addition, the cutoff for each LN status was optimized by maximizing the significance. Correlation analysis was performed using the Spearman rank correlation coefficient. Multivariate Cox proportional hazard regression analysis was developed using stepwise regression among representative variables, and the hazard radio (HR) and 95 % confidence interval (CI) for each variable were calculated. Furthermore, adequacy of the parametric model was assessed by the Akaike information criterion (AIC) [24] to estimate the most fitting model with the smallest information loss and to compare models consisting of various combinations of variables. Differences were considered statistically significant at the *p* < 0.05 level.

All statistical calculations were performed by EZR statistical software (Saitama Medical Center, Jichi Medical University, Saitama, Japan) [25], which is a graphical user interface for R (The R Foundation for Statistical Computing, Vienna, Austria).

## Results

**Fig. 2:**
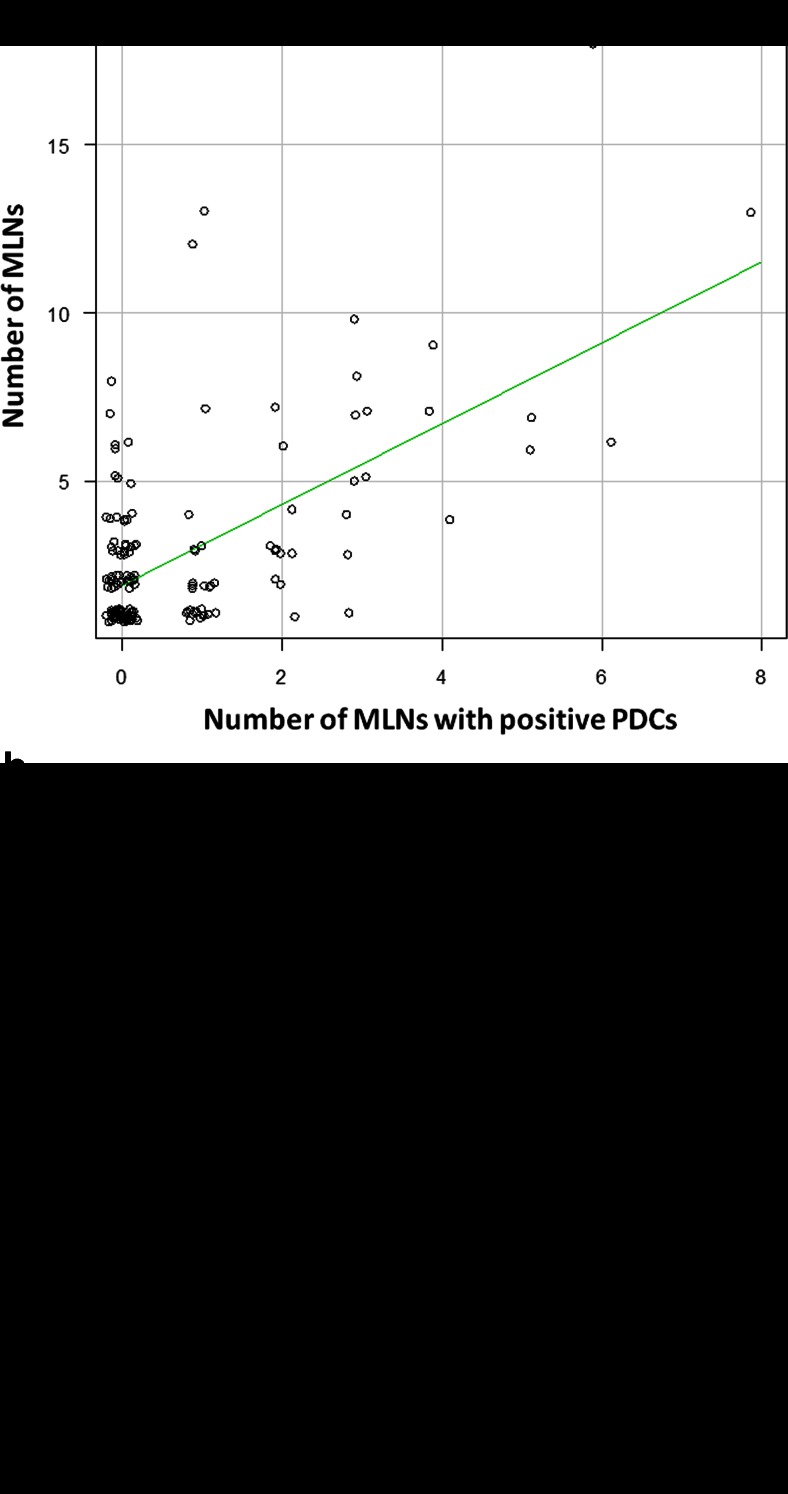
**a** Scatter plots for the number of MLNs-PDCs and the number of MLNs. The graph shows a moderate correlation between the two (Spearman rank correlation coefficient 0.405, *p* < 0.0001). **b** Scatter plots for the number of MLNs with positive and negative PDCs. Little correlation is found between the two (Spearman rank correlation coefficient 0.145, *p* = 0.068)

The study enrolled 159 patients. As Table 1 shows, 111 tumors (70 %) were colonic and 48 (30 %) were rectal. Most patients had pT3 or pT4 lesions (90 %), and half had positive-PDC primary tumors (50 %). One hundred seven patients (69 %) received adjuvant chemotherapy consisting mainly of 5-fluorouracil (5-FU); however, 52 (31 %) did not. At the time of this study, the median follow-up was 48.5 months and the 5-year OS rate of this cohort was 79 %. On average, there were 22.7 DLNs; of those, 2.8 MLNs and 1.0 MLN-PDCs were detected pathologically. No difference was found between open and laparoscopic surgery in the number of DLNs and MLNs (mean DLNs, 23.5 vs. 21.7, respectively; mean MLNs, 3.1 vs. 2.6, respectively). The study found a moderately positive correlation between the number of MLNs-PDCs and number of MLNs and a slight correlation between the number of MLNs with positive and negative PDCs. Figure 2 shows scatter plots for the number of MLNs-PDCs and MLNs.

**Table 1:** Patients and tumor characteristics (*n* = 159)

Variables	
Age (year) [mean (range, SD)]	65.2 (21–94, 11.9)
Sex [*n* (%)]	
Male	76 (55)
Female	83 (45)
Tumor location [*n* (%)]	
Colon	
Cecum-transverse	51 (32)
Descending-sigmoid	60 (38)
Rectum	48 (30)
Tumor size (mm) [mean (range, SD)]	47 (10–110, 19.4)
T status [*n* (%)]	
pT1	1 (1)
pT2	15 (9)
pT3	105 (66)
pT4	38 (24)
PDCs in primary tumor [*n* (%)]	
Positive	80 (50)
Negative	79 (50)
DLNs [mean (range, SD)]	22.7 (12–91, 11.7)
MLNs [mean (range, SD)]	2.8 (1–18, 2.7)
LNR [median (IQR)]	0.09 (0.06–0.17)
MLNs-PDCs [mean (range, SD)]	1.0 (0–8, 1.4)
Operative approach [*n* (%)]	
Open	83 (52)
Laparoscopic	76 (48)
Adjuvant chemotherapy [*n* (%)]	
With	107 (69)
Without	52 (31)

*PDCs* poorly differentiated clusters, *DLNs* dissected lymph nodes, *MLNs* metastatic lymph nodes, *LNR* lymph node ratio, *MLNs-PDCs* metastatic lymph nodes with positive PDCs

**Fig. 3:**
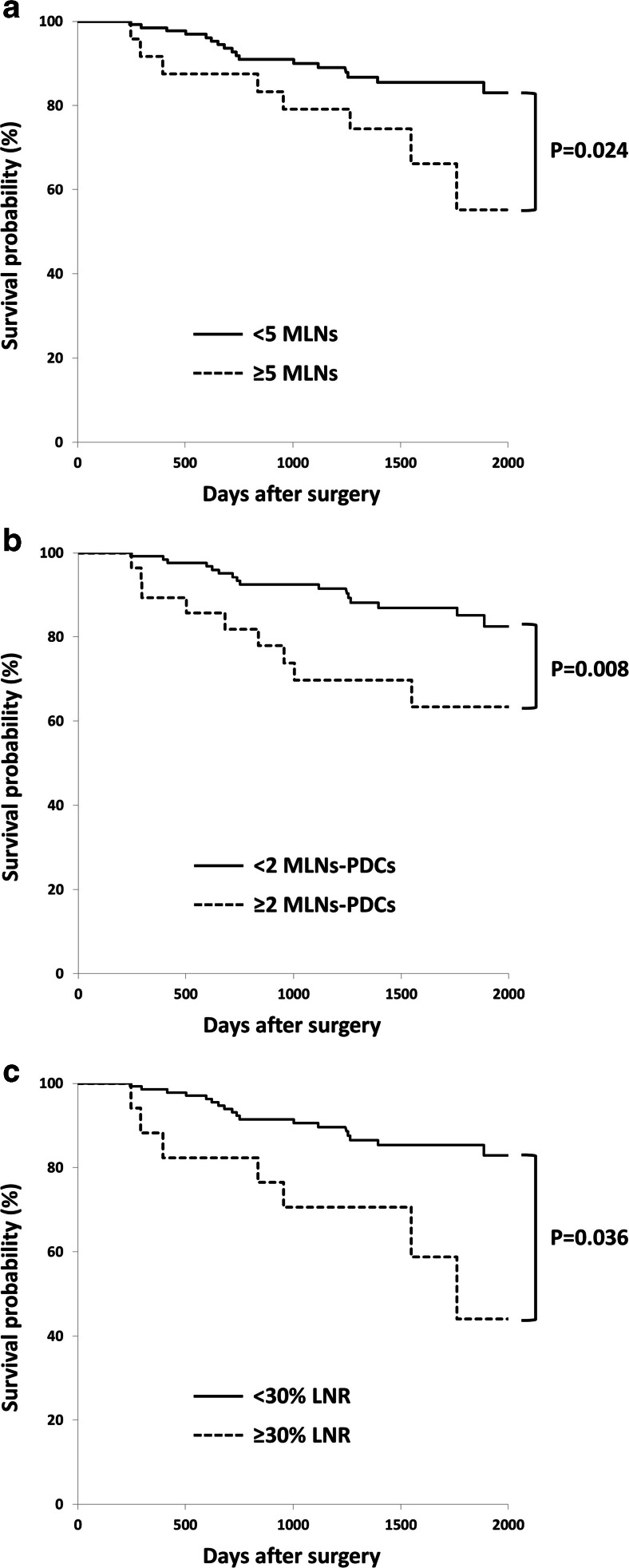
Comparison of survival curves for each lymph node status

Based on the Kaplan-Meier survival method, univariate analysis identified a cutoff of 5 MLNs (86 vs. 55 %, *p* = 0.024), 2 MLNs-PDCs (85 vs. 63 %, *p* = 0.008), and LNR of 30 % (85 vs. 44 %, *p* = 0.036) as the statistically reliable stratifications to discriminate patient survival (Fig. 3). Given this, we decided upon ≥5 MLNs, ≥2 MLNs-PDCs, and ≥30 % LNR to indicate positive LN status for subsequent analysis. On the other hand, when the study analyzed from 13 to 91 DLNs, a statistical threshold for 5-year OS was not obtained. Table 2 shows comparison of clinicopathological variables between patients with ≥2 MLNs-PDCs and <2. MLNs-PDCs (≥2) were associated with pT4 tumors (*p* = 0.0035), open surgery (*p* = 0.016), a greater number of MLNs (*p* < 0.0001), and a positive-PDC primary tumor (*p* < 0.0001). When the combination of PDC status in primary tumors and the number of MLNs-PDCs was examined, there were only two (3 %) negative-PDC primary tumors among those with ≥2 MLNs-PDCs. In contrast, of patients with <2 MLNs-PDCs, 53 (41 %) had positive-PDC primary tumors and 77 (59 %) had negative-PDC ones.

**Table 2:** Comparison of variables between patients with ≥2 and <2 MLNs-PDCs

Variables	≥2 MLNs-PDCs (n = 29)	<2 MLNs-PDCs (*n* = 130)	P value
Age (year) [mean (range, SD)]	66.5 (21–95, 14.0)	65.0 (21–85, 11.4)	
Sex [*n* (%)]			
Male	15 (10)	61 (38)	0.64
Female	14 (9)	69 (43)	
Tumor location [*n* (%)]			
Colon			0.11
Cecum-transverse	12 (8)	39 (25)	
Descending-sigmoid	6 (4)	54 (34)	
Rectum	11 (7)	37 (24)	
Tumor size (mm) [mean (range, SD)]	54 (20–110, 24.3)	45 (20–110, 18.0)	0.16
T status [*n* (%)]			
pT1	0 (0)	1 (1)	0.0035 (T1–3 vs. T4)
pT2	0 (0)	15 (9)	
pT3	16 (10)	89 (56)	
pT4	13 (8)	25 (16)	
PDCs in primary tumor [*n* (%)]			
Positive	27 (17)	53 (33)	<0.0001
Negative	2 (1)	77 (48)	
DLNs [mean (range, SD)]	23.9 (12–85, 14.4)	22.4 (12–91, 11.1)	0.68
MLNs [mean (range, SD)]	5.51 (2–18, 3.55)	2.19 (1–13, 1.95)	<0.0001
LNR [median (IQR)]	0.24 (0.13–0.37)	0.08 (0.05–0.14)	<0.0001
Operative approach [*n* (%)]			
Open	21 (13)	62 (39)	0.016
Laparoscopic	8 (5)	68 (43)	
Adjuvant chemotherapy [*n* (%)]			
With	22 (14)	85 (53)	0.28
Without	7 (4)	45 (28)	

Furthermore, in the subgroup analysis for the 128 patients with <5 MLNs, the 5-year OS rates of the patients with ≥2 (*n* = 14) and <2 MLNs-PDCs (*n* = 114) were 68 and 88 % (*p* = 0.037). In contrast, in subgroup analysis for the 26 patients with ≥5 MLNs, there was no significant difference in the 5-year OS rates between the patients with ≥2 and <2 MLNs-PDCs (59 and 53 %; *p* = 0.70). In addition, in another subgroup analysis for patients who received adjuvant chemotherapy and those who did not, 5-year OS rates of patients with and without chemotherapy were 67 vs. 64 % in ≥2 MLNs-PDCs and 87 vs. 82 % in <2 MLNs-PDCs, and no significant difference was found between the groups.

As Table 3 shows, among the representative clinicopathological variables, including LN status, the following five variables were identified using the AIC in multivariate analysis: tumor location (rectum vs. colon), number of MLNs-PDCs (≥2 vs. <2), sex (female vs. male), tumor size (≥50 vs. <50 mm), and T status (pT4 vs. pT1–3). Consequently, both pT4 tumors (HR = 3.54, *p* < 0.001) and ≥2 MLNs-PDCs (HR = 2.50, *p* = 0.038) were revealed as significant prognosticators for 5-year OS. Finally, the prognostic model incorporating ≥2 MLNs-PDCs had higher accuracy with lower AIC value (AIC = 230.08) than models incorporating other indicators of LN status, such as ≥5 MLNs (AIC = 231.21) or ≥30 % LNR (AIC = 231.77).

**Table 3:** Univariate and multivariate survival analysis of histological valuables

Variables	Univariate^a^	Multivariate^b^		
	*p* Value	HR	95 % CI	*p* Value
Age (year) (≥65 vs. <65)	0.29	–	–	–
Sex (female vs. male)	0.23	2.15	0.88–5.24	0.093
Tumor location (rectum vs. colon)	0.072	2.23	0.98–5.05	0.055
Tumor size (≥50 vs. <50 mm)	0.70	2.20	0.89–5.45	0.089
T status (T4 vs. T1–3)	0.032	3.54	1.37–9.14	0.009
MLNs (≥5 vs. <5)	0.024	–	–	–
LNR (≥30 vs. <30 %)	0.004	–	–	–
MLNs-PDCs (≥2 vs. <2)	0.008	2.50	1.09–5.74	0.031
Adjuvant chemotherapy (without vs. with)	0.31	–	–	–

^a^Kaplan and Meier method, and statistical significance was determined by log-rank test

^b^Multivariate survival analysis was performed using Cox’s proportional hazard model

*HR* hazard ratio, *CI* confidence interval

## Discussion

The present study examined prognostic properties of the number of MLNs-PDCs in a retrospective cohort comprising 159 patients with pStage III CRC. Since the clinical importance of PDCs in primary tumors has been clarified successfully [18, 19, 23, 26], criteria modified by the authors was applied to investigate histopathological features in MLNs. To our knowledge, this is the first study to highlight the histopathological features of PDCs in MLNs as a candidate for a new LN status.

Results showed that ≥2 MLNs-PDCs were associated closely with PDC-positive primary tumors, implying that PDC status in metastatic sites frequently could parallel that in primary tumors. On the other hand, this study found that some PDC-positive tumors were not accompanied by PDC-positive MLNs. Using similar approaches, Takahashi et al. [27] found histopathological heterogeneity between primary tumors and their synchronous MLNs in CRC patients. Furthermore, it is worth mentioning that Mori et al. [28] demonstrated that histopathological heterogeneity between primary tumors and their synchronous MLNs was associated with poorer survival even after adjuvant chemotherapy was administered. However, in the subgroup of this study that examined the effect of adjuvant chemotherapy, no significant difference was found between patients with ≥2 and <2 MLNs-PDCs. It is possible that this inconsistent result is due to the small sample size. Another possible explanation is that there was no control over the chemotherapy regimens given to patients, since those regimens progressed from single-dose 5-FU to combinations using 5-FU and oxaliplatin or irinotecan or both [2, 3] over the period of this study. In this context, additional prospective studies using larger sample sizes would confirm our results.

The most important finding of this study was that ≥2 MLNs-PDCs were associated strongly with a poorer 5-year OS. Although the number of MLNs-PDCs was correlated moderately positively with the number of MLNs, the number of MLNs-PDCs could provide the highest degree of discrimination regarding patient survival compared with the number of MLNs and other measures of LN status that are based on the theory of AIC. These findings suggest that the number of MLNs-PDCs could provide more precise information on potential tumor aggressiveness at the metastatic site. Furthermore, this study assessed the subgroups of patients with <5 MLNs, and there was significant difference in the 5-year OS rates between patients with ≥2 and <2 MLNs-PDCs. Consequently, this outcome suggests that MLNs-PDCs might contribute to further survival stratification, especially for CRC patients with <5 MLNs. Interestingly, Komuta et al. [29] and Fujii et al. [30] reported the “extracapsular invasion” of MLNs, which was highly indicative of tumor aggressiveness at the metastatic site. This concept seems to be similar to that of PDCs in MLNs in our study. Likewise, Ueno et al. [16] described EX that exhibited active, invasive lesions as “aggressive EX,” which was associated with a dramatically worse prognosis. Since evaluation criteria in this study allowed EX to be comprehensively considered as MLNs, it is possible that MLNs with positive PDCs included some aggressive EX.

This study has some potential limitations. First, the 5-year OS rate of 79 % in patients with pStage III CRC in this cohort seems to be relatively better than that estimated in studies with larger populations [2, 3]. The reason for this remains unclear. Second, the exceedingly high hazard ratio may be a product of the single-institution experience and the relatively small sample size in this study. Another question is why ≥2 MLNs-PDCs were related to open surgery (*p* = 0.016) even though the number of DLNs and MLNs did not differ statistically between open and laparoscopic surgery. One possible explanation is surgeons’ preoperative choices, in which cases with clinically diagnosed T4 tumors were treated by open surgery. In addition, although cumulative evidence has confirmed a larger number of MLNs as a strong prognosticator of CRC, cutoff thresholds for some LN statuses addressed in this study remain under intense discussion. With respect to the cutoff threshold of LNR, Yuan et al. [13] defined it as 33 %, Gleisner et al. [14] as 50 %, and Madbouly et al. [15] as 38 %. Although consensus has not been reached regarding this cutoff, they agreed that a higher LNR was an adverse prognosticator. The cutoff of 30 % in this study seems to be comparable to aforementioned data. In contrast, similar to the experience of Kotake et al. [10], we did not identify a statistically reliable cutoff for the number of DLNs. Although existing guidelines [5, 6] recommend that ≥12 DLNs be examined to eliminate possible staging migration, some researchers claim that there is no minimum number of DLNs that would enhance the accuracy of staging pStage III CRC [8, 9]. Therefore, this issue merits further discussion.

## Conclusion

The PDCs in MLNs were closely associated with that in primary tumors. The number of MLNs-PDCs could exert an effect on survival and provide more concise information to improve stratification in pStage III CRC and, consequently, could be a candidate for a new, alternative LN status. This study demonstrated the need for special observation of PDCs in MLNs during pathological diagnosis.

## References

[1] Ferlay J, Shin HR, Bray F (2010). Estimates of worldwide burden of cancer in 2008: GLOBOCAN 2008. Int J Cancer.

[2] Hurwitz H, Fehrenbacher L, Novotny W (2004). Bevacizumab plus irinotecan, fluorouracil, and leucovorin for metastatic colorectal cancer. N Engl J Med.

[3] Fuchs CS, Marshall J, Mitchell E (2007). Randomized, controlled trial of irinotecan plus infusional, bolus, or oral fluoropyrimidines in first-line treatment of metastatic colorectal cancer: results from the BICC-C Study. J Clin Oncol.

[4] Dukes C (1937). Histological grading of rectal cancer: (section of pathology). Proc R Soc Med.

[5] Leslie HS, Mary KG, Christian W (2009) TNM classification of malignant tumours, 7th edition, Wiley-Blackwell

[6] Japanese Society for Cancer of the Colon and Rectum (2013). Japanese classification for colorectal carcinoma.

[7] Tepper JE, O'Connell MJ, Niedzwiecki D (2001). Impact of number of nodes retrieved on outcome in patients with rectal cancer. J Clin Oncol.

[8] Goldstein NS (2002). Lymph node recoveries from 2427 pT3 colorectal resection specimens spanning 45 years: recommendations for a minimum number of recovered lymph nodes based on predictive probabilities. Am J Surg Pathol.

[9] Chou JF, Row D, Gonen M (2010). Clinical and pathologic factors that predict lymph node yield from surgical specimens in colorectal cancer: a population-based study. Cancer.

[10] Kotake K, Honjo S, Sugihara K (2012). Number of lymph nodes retrieved is an important determinant of survival of patients with stage II and stage III colorectal cancer. Jpn J Clin Oncol.

[11] Berger AC, Sigurdson ER, LeVoyer T (2005). Colon cancer survival is associated with decreasing ratio of metastatic to examined lymph nodes. J Clin Oncol.

[12] Kobayashi H, Mochizuki H, Kato T (2011). Lymph node ratio is a powerful prognostic index in patients with stage III distal rectal cancer: a Japanese multicenter study. Int J Colorectal Dis.

[13] Yuan Y, Li MD, Hu HG (2013). Prognostic and survival analysis of 837 Chinese colorectal cancer patients. World J Gastroenterol.

[14] Gleisner AL, Mogal H, Dodson R (2013). Nodal status, number of lymph nodes examined, and lymph node ratio: what defines prognosis after resection of colon adenocarcinoma?. J Am Coll Surg.

[15] Madbouly KM, Abbas KS, Hussein AM (2014). Metastatic lymph node ratio in stage III rectal carcinoma is a valuable prognostic factor even with less than 12 lymph nodes retrieved: a prospective study. Am J Surg.

[16] Ueno H, Mochizuki H, Hashiguchi Y (2007). Extramural cancer deposits without nodal structure in colorectal cancer: optimal categorization for prognostic staging. Am J Clin Pathol.

[17] Ueno H, Mochizuki H, Shirouzu K (2012). Multicenter study for optimal categorization of extramural tumor deposits for colorectal cancer staging. Ann Surg.

[18] Ueno H, Kajiwara Y, Shimazaki H (2012). New criteria for histologic grading of colorectal cancer. Am J Surg Pathol.

[19] Ueno H, Hase K, Hashiguchi Y (2014). Site-specific tumor grading system in colorectal cancer: multicenter pathologic review of the value of quantifying poorly differentiated clusters. Am J Surg Pathol.

[20] Barresi V, Branca G, Vitarelli E (2014). Micropapillary pattern and poorly differentiated clusters represent the same biological phenomenon in colorectal cancer: a proposal for a change in terminology. Am J Clin Pathol.

[21] Sugihara K, Kobayashi H, Kato T (2006). Indication and benefit of pelvic sidewall dissection for rectal cancer. Dis Colon Rectum.

[22] Akiyoshi T, Watanabe T, Miyata S (2012). Results of a Japanese nationwide multi-institutional study on lateral pelvic lymph node metastasis in low rectal cancer: is it regional or distant disease?. Ann Surg.

[23] Kinoshita O, Kishimoto M, Murayama Y (2015). Semi-quantified pathological evaluation of poorly differentiated clusters in non-mucinous pT2-3 colorectal carcinoma. J World Surg Oncol.

[24] Akaike H (1974). A new look at the statistical model identification. IEEE Trans Autom Control.

[25] Kanda Y (2013). Investigation of the freely available easy-to-use software 'EZR' for medical statistics. Bone Marrow Transplant.

[26] Barresi V, Bonetti LR, Ieni A (2014). Histologic grading based on counting poorly differentiated clusters in preoperative biopsy predicts nodal involvement and pTNM stage in colorectal cancer patients. Hum Pathol.

[27] Takahashi K, Mori T, Yasuno M (2000). Histologic grade of metastatic lymph node and prognosis of rectal cancer. Dis Colon Rectum.

[28] Mori T, Hirota T, Ohashi Y (2006). Significance of histologic type of primary lesion and metastatic lymph nodes as a prognostic factor in stage III colon cancer. Dis Colon Rectum.

[29] Komuta K, Okudaira S, Haraguchi M (2001). Identification of extracapsular invasion of the metastatic lymph nodes as a useful prognostic sign in patients with resectable colorectal cancer. Dis Colon Rectum.

[30] Fujii T, Tabe Y, Yajima R et al (2011) Process of distant lymph node metastasis in colorectal carcinoma: implication of extracapsular invasion of lymph node metastasis. BMC Cancer 11:216. doi:10.1186/1471-2407-11-216 10.1186/1471-2407-11-216 PMC3118198 21635742

